# Polygenic Risk Score Analysis of Alzheimer's Disease in Cases without APOE4 or APOE2 Alleles

**DOI:** 10.14283/jpad.2018.46

**Published:** 2018-12-14

**Authors:** V. Escott-Price, A. Myers, M. Huentelman, M. Shoai, John Hardy

**Affiliations:** 1 Dementia Research Institute, MRC Centre for Neuropsychiatric Genetics and Genomics, Cardiff University, Cardiff, UK; 2 Department of Psychiatry & Behavioral Sciences, Programs in Neuroscience and Human Genetics and Genomics and Center on Aging, Miller School of Medicine, University of Miami, Miami, FL, USA; 3 Neurogenomics Division, The Translational Genomics Research Institute (TGen), 85004, Phoenix, AZ, USA; 4 Department of Molecular Neuroscience and Reta Lilla Weston Laboratories, Institute of Neurology, London, UK

**Keywords:** Alzheimer's disease, genetics, pathology, APOE

## Abstract

The We and others have previously shown that polygenic risk score analysis (PRS) has considerable predictive utility for identifying those at high risk of developing Alzheimer's disease (AD) with an area under the curve (AUC) of >0.8. However, by far the greatest determinant of this risk is the apolipoprotein E locus with the E4 allele alone giving an AUC of ∼0.68 and the inclusion of the protective E2 allele increasing this to ∼0.69 in a clinical cohort. An important question is to determine how good PRS is at predicting risk in those who do not carry the E4 allele (E3 homozygotes, E3E2 and E2E2) and in those who carry neither the E4 or E2 allele (i.e. E3 homozygotes). Previous studies have shown that PRS remains a significant predictor of AD risk in clinical cohorts after controlling for APOE ε4 carrier status. In this study we assess the accuracy of PRS prediction in a cohort of pathologically confirmed AD cases and controls. The exclusion of APOE4 carriers has surprisingly little effect on the PRS prediction accuracy (AUC ∼0.83 [95% CI: 0.80-0.86]), and the accuracy remained higher than that in clinical cohorts with APOE included as a predictor. From a practical perspective this suggests that PRS analysis will have predictive utility even in E4 negative individuals and may be useful in clinical trial design.

## Introduction

**P**olygenic risk score (PRS) analysis enhances the predictability of the diagnosis of AD (1). In a recent analysis, we showed that the area under the curve (AUC) in a pathologically confirmed case/control series was 0.84 (2). However, by far the largest contribution to this risk analysis is the E4 allele (risk) and the E2 allele (protective) which gave AUC of 0.68 (E4 alone) and 0.69 (E4+E2) as compared to the overall PRS AUC=0.75 in clinical samples (1). An important practical and theoretical consideration is to understand how good PRS is when the risk at the APOE locus is removed. When this was tested in the clinical series (1) the AUC was reduced from 0.75 in the whole dataset to 0.65 in E3 homozygotes. Assessment of the significance of PRS adjusting for APOE4 statistically was performed (3) and indicated little change in the models' statistical significance. However for practical application, e.g. selecting individuals for clinical trials, statistical significance is not an informative measure of the algorithm performance. To our knowledge, the PRS accuracy in E3 homozygotes has never been directly investigated in pathologically confirmed samples. Therefore, we tested this in our pathological series by removing from the analysis, first all E4 carriers and then, all E4 and E2 carriers from both the case and the control data sets.

The sample characteristics of the original dataset used in this study were the same as in our previous analysis (2). This project was declared IRB exempt (MedstarProject #2003-118) under the Code of Federal Regulations, 45 CFR, 46. The primary data consisted of 1011 cases and 583 controls. We first eliminated all those samples who had an E4 allele (leaving 354 cases and 454 controls) and then additionally those with an E2 allele (leaving 321 cases and 365 controls homozygous for the E3 allele). From the total 36,481,940 imputed single nucleotide polymorphisms (SNPs), we excluded those with an Info score below 0.8 and MAF<0.01. This resulted in 7,868,100 SNPs which were used for the analysis. Genome-wide association analysis was performed for each SNP using logistic regression analysis as implemented in snptest (4) with adjusting for gender and first two principal components which were selected after visual inspection of each pair of PCs to adjust foe any potential stratification in the data

Predictive modelling was performed using a polygenic score approach based upon AD associated SNPs according to the IGAP study (5). We converted the imputed genotypes of our samples into “most probable” genotypes with a probability over 90%. The correlated SNPs were pruned using parameters r2=0.1, a physical distance threshold of 500Kb, preferentially retaining the SNP most significantly associated with AD (2). The AD GWAS association p-value threshold for SNP inclusion was 0.5, as this currently maximally captures polygenic risk in the greatest number of samples (1). The models were fitted using IGAP (stage I) summary statistics data as a training set and predicting AD/control status in our study. We note that our cohort is part of the IGAP study (5) and therefore the results maybe slightly biased due to the 1.3% overlap. To adjust for the overlap, we used a simulation approach as described in (2), assuming that our dataset (N=686) is a random subset of the IGAP study (N=54,162). In short, we simulated 1000 times effect sizes of SNPs with mean b∼N(BIGAP, sd=0.12*SEIGAP), where BIGAP is the beta-coefficient and SEIGAP is the standard error for that SNP in the IGAP study, and the coefficient 0.12 was estimated empirically (see (2) for details).

## Results

The prediction accuracy (AUC) in the full pathologically confirmed dataset was AUC=0.73 [0.71-0.75] for E4 alleles and AUC= 0.75 [0.73-0.77] for E4 and E2. When PRS was included to the predictive model, the AUC for the full pathologically confirmed dataset was 0.84, after adjusting for the overlap with the training IGAP dataset used for SNP selection (2). The original unadjusted AUC was 0.87 and was 0.84, after adjusting for the overlap with the training set used for SNP selection (2).

Removing all individuals with an E4 allele only reduced the unadjusted AUC from 0.87 to 0.84 and then removing all E2 carriers (i.e. restricting the analysis to E3 homozygotes) had a further small effect and reduced the AUC to 0.83. Thus, in contrast to the results obtained with the clinical series, the AUC is only marginally reduced by removing E4 and E2 carriers.

We tested three possible explanations for this finding: 1) the people who get AD without an E4 allele have more AD risk alleles, i.e. alleles at other loci have bigger effects in the absence of E4; 2) the effects of APOE and other risk SNPs are independent; 3) the results are driven by inflation due to the overlap between the discovery (IGAP) and test (E3 homozygote pathologically confirmed AD cases and controls) datasets.

First, we ran a GWAS analysis with snptest software only for E3 homozygote cases and controls. The majority of the top IGAP SNPs did not show statistically significant association in this small sample set and their effect sizes were not higher than the effect sizes in the whole data set (data not shown). This strongly suggests that the E3 homozygotes with disease do not have a greater excess of other AD risk alleles.

Next, we counted the number of risk and alternative alleles for sets of SNPs at different significance thresholds (reported by the IGAP study) for each subject in the E3 homozygote subgroup and in the rest of the dataset. We compared the average number of risk and alternative alleles (per person) using a chi-square test for a 2x2 table: (Risk Allele - Alternative Allele) x (E3 homozygotes – other genotypes). This analysis was performed in cases and controls separately as cases in general may have more risk alleles than controls. The results are summarized in Tables 1 and 2, respectively. There were no significant differences in the mean number of risk and alternative alleles per person among the E3 homozygotes versus the other genotypes in either the pathologically confirmed AD cases or the pathologically confirmed controls.

**Table 1:** Comparison of the mean numbers of risk and alternative alleles per person in E3 homozygotes vs other AD cases. APOE region is excluded

**SNP selection threshold**	**E3 homozygotes**		**Other genotypes**		**OR**	**P**
	**Mean No of risk alleles**	**Mean No of alternative alleles**	**Mean No of risk alleles**	**Mean No of alternative alleles**		
0.0001	92.5	63.3	91.0	63.6	1.016	1
0.001	306.6	282.2	299.9	283.8	1.022	0.859
0.01	1777.9	1576.4	1766.1	1580.1	1.007	0.874
0.05	7348.8	6333.3	7315.1	6346.3	1.005	0.794
0.1	13329.7	11633.7	13300.3	11661.5	1.002	0.805
0.2	23935.0	21032.8	23868.4	21084.1	1.003	0.701
0.3	33149.4	29575.3	33081.5	29657.7	1.002	0.673
0.4	41612.3	37349.4	41561.4	37436.3	1.001	0.729
0.5	49267.5	44544.9	49197.0	44643.4	1.001	0.697

**Table 2:** Comparison of the mean numbers of risk and alternative alleles per person in E3 homozygotes vs other controls. APOE region is excluded

**SNP selection threshold**	**E3 homozygotes**		**Other genotypes**		**OR**	**P**
	**Mean No of risk alleles**	**Mean No of alternative alleles**	**Mean No of risk alleles**	**Mean No of alternative alleles**		
0.0001	87.6	66.8	88.4	67.7	0.991	1.00
0.001	293.8	293.2	292.8	295.4	1.003	0.973
0.01	1727.4	1621.3	1723.0	1623.0	1.003	0.961
0.05	7181.6	6461.3	7153.8	6471.3	1.004	0.833
0.1	13070.4	11836.9	13037.7	11860.3	1.003	0.810
0.2	23540.4	21344.5	23466.5	21388.0	1.003	0.703
0.3	32649.2	29969.2	32573.0	30039.3	1.002	0.684
0.4	41028.5	37805.2	40983.2	37869.4	1.001	0.785
0.5	48618.4	45048.2	48564.7	45118.6	1.001	0.776

We also compared the predictive accuracy of the best model (PRS for SNPs with p-values≤0.5) with and without APOE in three subgroups, namely E4 carriers (644 cases and 115 controls), E4 and E2 carriers (677 cases and 204 controls) and E3 homozygotes (321 cases and 365 controls). Table 3 shows the estimated AUC in those subgroups for the PRS models with more significant SNPs (p≤0.001 in IGAP study) and the best predictive PRS model (1), combining all available independent SNPs with p-values ≤0.5, when the APOE region is included and excluded. The results clearly show that the PRS predictive accuracy is almost the same in any subgroup, when APOE is excluded. Note that the full dataset (shown in the second column of Table 3), has the largest overlap with IGAP, and therefore the AUC estimate for this group has the most (∼2%) inflation (see (2) for details).

**Table 3:** AUC for PRS models with IGAP-based p-value SNP selection thresholds 0.001 and 0.5. These results are not unadjusted for IGAP/Corneveaux overlap

**PRS model**	**AUC and 95% Confidence intervals in “[]”**			
	**whole sample**	**E4 carriers**	**E4E2 carriers**	**E3 homozygotes**
PRS with SNPs p≤0.001	0.741 [0.72-0.78]	0.616 [0.56-0.67]	0.743 [0.70-0.78]	0.632 [0.59-0.67]
PRS with SNPs p≤0.5	0.866* [0.85-0.89]	0.831 [0.78-0.88]	0.868 [0.84-0.90]	0.831 [0.80-0.86]
PRS with SNPs p≤0.001 APOE region excluded	0.637 [0.61-0.67]	0.565 [0.51-0.62]	0.625 [0.58-0.67]	0.646 [0.61-0.69]
PRS with SNPs p≤0.5 APOE region excluded	0.840 [0.82-0.86]	0.821 [0.77-0.87]	0.837 [0.80-0.87]	0.834 [0.80-0.86]

Finally, we adjusted our main result (AUC=0.83 in the E3 homozygote dataset) for the overlap with the discovery IGAP dataset using a simulation approach (2). The adjusted AUC and the confidence intervals were calculated as average AUC and CI over 1000 simulations, AUCADJ = 0.83 [95% CI: 0.80-0.86].

**Figure 1:**
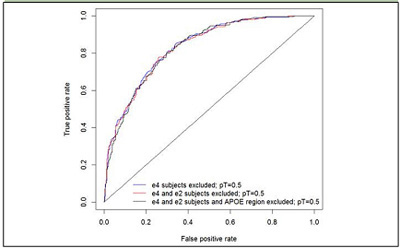
Polygenic Risk Score with E4 allele carriers omitted and in E3 homozygotes

**Figure 2:**
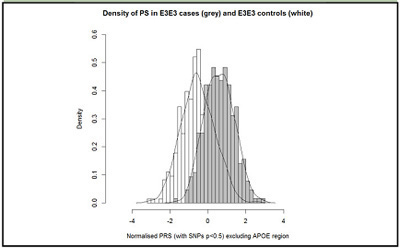
Distribution of standardised and PCA adjusted PRS in E3E3 cases and controls

Figure 2 shows the distribution of standardized PRS for the E3 homozygote cases and E3 homozygote controls. In the negative polygenic extreme group (PRS smaller than -2), there were 17 controls and 0 cases. In the positive extreme group (PRS greater than 2), there were 11 cases and 1 control. Looking at the extremes (PRS < -1.5) and (PRS > 1.5), there were 1 case and 49 controls and 41 cases and 4 controls, respectively.

## Discussion

Our results show that the predictive accuracy of PRS in pathologically confirmed E3 homozygotes is high and equivalent to the predictive accuracy of the whole dataset. This finding indicates that APOE is an independent risk factor for the disease. This result is in contrast to the PRS observed in clinical cohorts where restricting analyses to E3 homozygotes resulted in a large reduction in the PRS accuracy. We believe this is likely to be because of poor diagnostic accuracy among those labeled as AD in the absence of an E4 allele: this interpretation is consistent with post mortem follow up of AD clinical trials, which suggested a diagnostic inaccuracy of up to 25% (6, 7). From a mechanistic perspective, this result suggests that the genetic architecture of AD in E3 homozygotes is similar to that in the other APOE genotypes since a similar proportion of risk is captured by PRS in all genotypes. This result does not support the belief that E3 homozygotes with AD have more predisposing variants at other loci. This result suggests that PRS analysis is likely to have utility in clinical trial design.

*Acknowledgements:* This manuscript is dedicated to the memory of our colleagues who worked on generating these data:- Christopher B. Heward and Jason J. Corneveaux. We thank the patients and their families for their selfless donations. The data generation for this project was supported by funding from Kronos Science. Additional funding was from the National Institutes of Health as well as NIH EUREKA grant R01-AG-034504 to AJM and AG041232 (NIA) to AJM and MH as well as Intramural funds NIH (JH and AJM). Analytical work was supported the MRC JPND PERADES grant MR/L501517/1 (JH and VEP). Many data and biomaterials were collected from several National Institute on Aging (NIA) and National Alzheimer's Coordinating Center (NACC, grant #U01 AG016976). A full listing off collection sites is given in ref. 4. Professors Hardy and Escott Price are members of the UKDRI. JH is supported by the Dolby Foundation, and by the National Institute for Health Research University College London Hospitals Biomedical Research Centre

*Author contributions:* VEP carried out the PRS analysis. AM and MH generated the original data and quality controlled it for this analysis. JH designed the study and wrote the original draft. All authors obtained funds for the study and analysis and reviewed the drafts.

*Potential Conflict of Interest:* JH and VEP are a co-grantees of Cytox from Innovate UK (UK Department of Business).
